# First clinical pregnancy and delivery achieved after using a new 3D imaging technology for sperm selection: a case report

**DOI:** 10.3389/frph.2025.1559684

**Published:** 2025-03-07

**Authors:** Yulia Michailov, Shevach Friedler, Bozhena Saar-Ryss

**Affiliations:** ^1^ Obstetrics and Gynecology Department, Barzilai University Medical Center, Ashkelon, Israel; ^2^ Faculty of Health Sciences, Ben Gurion University of the Negev, Beer Sheva, Israel

**Keywords:** ART, OTA, sperm selection, ICSI, blastocyst

## Abstract

**Objective:**

To report a case of a patient who, after experiencing recurrent failed implantations, underwent a novel technological intervention—using the Q300 device—which successfully led to a pregnancy and delivery of a healthy baby.

**Design:**

Case report.

**Setting:**

Barzilai University Medical Center.

**Patient (s):**

A 33-year-old woman with primary infertility experienced recurrent implantation failure (RIF), while her 32-year-old male partner was diagnosed with severe oligo-astheno-teratozoospermia (OTA) syndrome.

**Intervention:**

Using Q300 device for selection of the morphologically compliant sperm cells for intracytoplasmic sperm injection (ICSI).

**Main outcome measures:**

Successful pregnancy and delivery.

**Results:**

A unique case of clinical pregnancy and delivery involving a couple facing RIF and severe OTA. In this case, a new technology for sperm selection was used. The semen sample was examined using the Q300 device to choose WHO2021-morphologically compliant sperm cells for micro-injection. The resulting embryos were developed and then frozen. Later, a frozen-thawed embryo transfer was performed during the following natural menstrual cycle, leading to successful pregnancy and delivery.

**Conclusion:**

The utilization of this new 3D imaging technology underscores the evolving landscape of reproductive medicine and the potential it holds for transforming outcomes in challenging cases. By documenting such cases, we contribute to the ongoing dialogue to refine assisted reproductive technology (ART) protocols and improve reproductive outcomes for individuals facing similar challenges.

**Trial registration:**

NCT06232720 https://clinicaltrials.gov/study/NCT06232720. Date of registration: 15 Feb 2023. Date of enrollment of the first subject: 20 August 2023.

## Introduction

The successful establishment of pregnancy following assisted reproductive technologies (ART) is a complex interplay of various factors, prominently including embryo quality (1) and endometrial receptivity (2). Despite advancements in reproductive medicine, cases of failed implantation remain a challenging scenario encountered by clinicians and patients alike. Failed implantation is defined as the inability of a transferred embryo to implant and progress to a clinical pregnancy despite apparently favorable conditions.

Failed implantation, characterized by the inability of an embryo to implant despite optimal conditions, poses significant emotional and clinical hurdles for both patients and clinicians (3). Addressing this challenge requires a comprehensive assessment of factors influencing embryo viability and endometrial receptivity.

Several studies emphasize the crucial role of sperm factors in recurrent implantation failure (RIF), highlighting the impact of sperm DNA integrity, oxidative stress levels, and other molecular attributes on embryo development and implantation success (4, 5). Although assisted reproductive technologies have advanced, embryologists continue to encounter significant challenges in manually selecting a good viability sperm cell (6). Conventional sperm selection methods based on subjective “manual” assessments of morphology and motility, do not always reflect genetic quality, potentially leading to sperm selection with DNA fragmentation or epigenetic abnormalities (7). This limitation highlights the necessity for quantitative, objective selection techniques, such as those based on advanced imaging technologies, to improve accuracy and optimize clinical outcomes.

This case report documents a compelling instance where a couple suffering from RIF (recurrent implantation failure) and a severe Oligo-Astheno-Teratozoospermia (OTA) syndrome of the male partner, achieved successful pregnancy and delivery through when assisted by a novel technological intervention—the Q300 device (QART Medical, Raanana, Israel) for 3D morphology assessment and selection of WHO2021 morphologically compliant sperm cells for intracytoplasmic sperm injection (ICSI).

**Figure 1:**
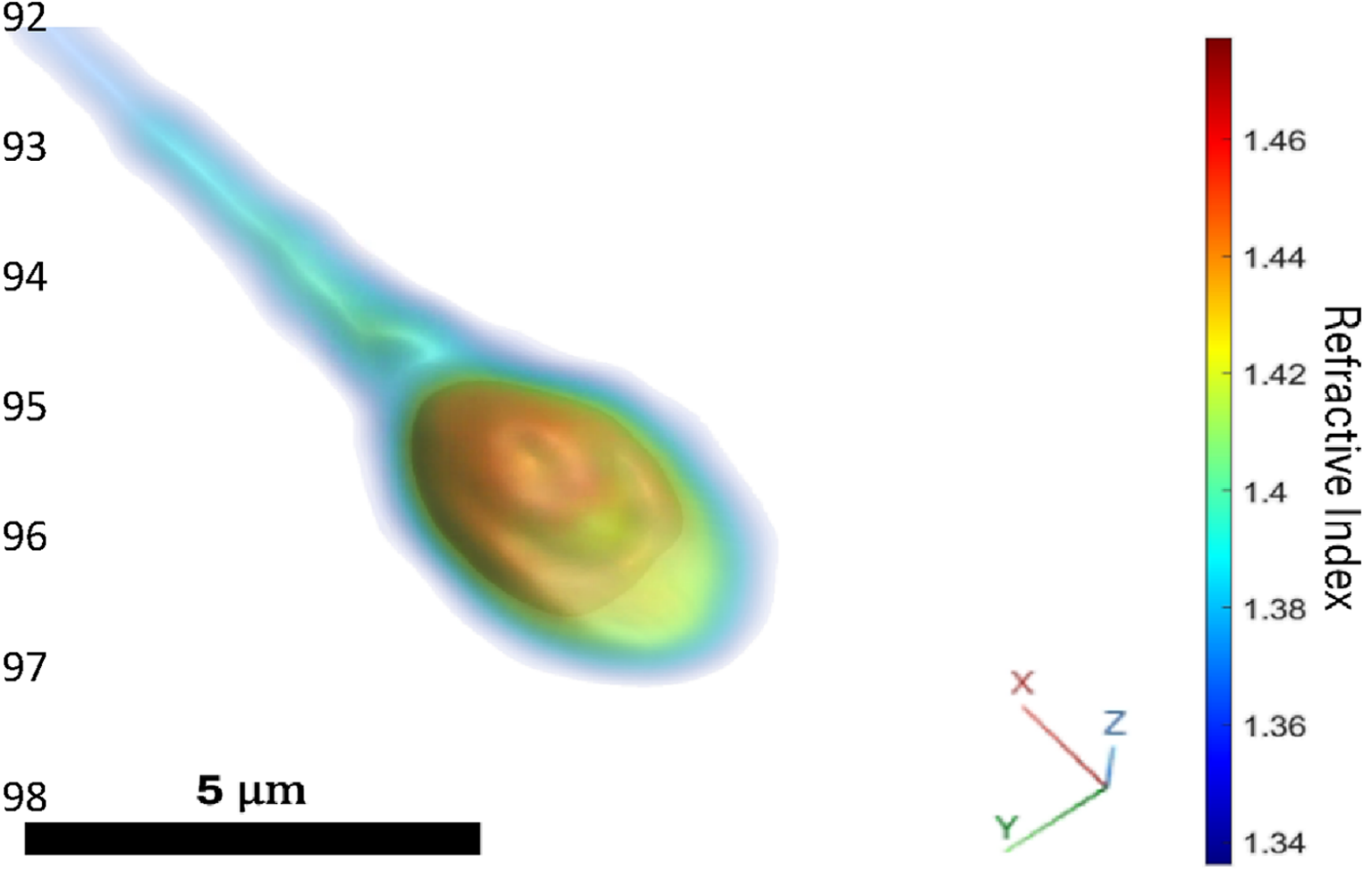
Volume rendering of the 3D refractive index distribution of one of the sperm injected in the ICSI procedure resulting in one of the two returned embryos. Blue region of low refractive index indicates cellular membrane, midpiece, and tail, yellow region of middle-range refractive index indicates acrosome and cytoplasm, red region of high refractive index indicates nucleus.

The Q300 device is an advanced optical imaging system designed for 3D morphological analysis of human sperm cells. This technology employs quantitative phase microscopy based on holographic imaging, enabling precise 3D refractive index measurement of live and motile sperm cells (8–10). The device images live, and motile sperm cells intended for subsequent injection into an oocyte for fertilization in ICSI procedures and provides users with an automatic, objective, and recorded 3D morphological analysis of each individual sperm cell (Figure 1) that the embryologist previously determined as normal and elected to inspect further. The device allows virtual staining, hence, label-free sperm visualization as if the cell is chemically stained, but without using actual staining. The morphological assessment is compared in real-time to World Health Organization (WHO) laboratory manual for the examination and processing of human semen criteria (11) that are otherwise restricted to semen sample morphological analysis, due to their destructive effect on the sperm cells. While the Q300 device aids the clinical embryologist in selecting sperm cells, the embryologist is making the final decision. This intervention aims to develop superior-quality embryos and achieve a successful pregnancy and live birth.

This introduction sets the stage by emphasizing the persistence of failed implantation despite existing technologies, the introduction of a new technology, and the potential impact on clinical practice and patient outcomes.

## Case presentation

The couple, a 33-year-old nulligravida (G0P0) woman and her 32-year-old male partner, both with unremarkable medical histories, were under the care of the IVF Unit at Barzilai University Medical Center, Ashkelon, Israel, for three years. They presented with primary infertility attributed to severe male factor. Despite undergoing 15 IVF cycle attempts, they have not achieved a successful conception. The female patient underwent an extensive evaluation to identify potential underlying causes of Recurrent Implantation Failure (RIF). This included a Transvaginal 3D Ultrasound and hysteroscopy with endometrial biopsy to exclude chronic endometritis. Additionally, hormonal and metabolic assessments were performed, including thyroid function tests (TSH and T4), prolactin and vitamin D levels. The patient also underwent antiphospholipid antibody testing, a thrombophilia panel, and karyotyping. All results were found to be within normal limits. The male partner underwent a comprehensive infertility assessment, which included a urological evaluation with physical examination and testicular ultrasound. A hormonal profile and karyotype analysis were also performed, with all results within normal limits.

The patients' 16th IVF treatment cycle was done using a Stop Gonadotropin-releasing hormone GnRH -agonist/GnRH-antagonist protocol (12). Due to suspected high risk of ovarian hyperstimulation syndrome (OHSS), it was decided to freeze all the embryos to avoid OHSS and to minimize possible adverse effects of hyperstimulation on the endometrium.

Ovum pick-up (OPU) of the patient was done on April 02, 2024. 13 oocytes were retrieved, 11 of them were MII. The men's sperm count was 3 million/ml, with 33% progressive motility and 0% normal morphology, low-quality sperm, according to WHO criteria (11). The semen sample was imaged by the Q300 device before micro-injection to aid in selecting morphologically compliant sperm cells. 11 sperm cells (according to embryologist selection and WHO criteria) were selected for injection into 11 MII oocytes. 8 zygotes were observed, and two blastocysts grade 4AA and G2 on day 5 according to the Gardner system (13), were formed and cryopreserved.

The embryo transfer was performed during the subsequent natural menstrual cycle, resulting in a positive pregnancy for the first time following multiple treatment cycles.

The patient's identifiable health information in this case has been anonymized for publication. Consent was obtained from the patients for their participation in this report's publication.

## Materials and methods

The 16th IVF treatment cycle was done using a Stop GnRH Agonist combined with a Multiple-Dose GnRH-Antagonist protocol as described by Orvieto (12). The patient received triptorelin acetate (Decapeptyl® Ferring pharmaceuticals Ltd, Netanya, Israel)0.1 mg/day, started in the mid-luteal phase and discontinued with the onset of menses and after confirmation of down-regulation by serum E2 levels and vaginal ultrasound measurement. Gonadotropins were initiated after two wash-out days with a daily dose of 200 IU of Pergoveris (Pergoveris®, Merck KGaA, Darmstadt, Germany, containing a fixed combination of r-hFSH and r-hLH at a ratio of 2:1) for 4 days and were substituted to 225 IU of Menopur (Highly Purified Human Menopausal Gonadotropin. Ferring pharmaceuticals Ltd, Netanya, Israel). Co-treatment with GnRH antagonist (0.25 mg/day, Cetrorelix, Cetrotide, Merck, Darmstadt, Germany) was started on day 5 of stimulation when E2 levels were 323 pg/ml. All the medications were continued until the triggering day. Peak serum 17-*β*-estradiol concentration was 5424 pg/ml on the day of the trigger; therefore, due to suspected high risk to develop OHSS, it was decided to trigger final follicular maturation with GnRH -agonist (triptorelin 0.2 mg) and freeze all the embryos.

During the OPU, 13 oocytes were retrieved, of which 11 were recognized as MII oocytes suitable for ICSI. Each sperm cell selected by the embryologist was imaged using the Q300 device before microinjection to ensure morphological compliance. Initially, 22 sperm cells were selected based on progressive motility and morphology. These cells were then imaged by the Q300 device to assess their morphological compliance according to WHO 2021 criteria (11).

Out of the 22 selected sperm cells, only 7 met the WHO 2021 morphological criteria, while 4 had borderline measurements, falling within 10% of the recommended standards. This resulted in 11 cells being compliant or borderline, and these were subsequently microinjected by electrohydraulic injectors (RI Integra micromanipulator, Cooper Surgical, Cooper Surgical, USA). Non-compliant sperm cells were discarded.

The oocytes were cultured in an Embryoscope by using Continuous Single Culture-NX Complete medium (CSCM -NXC) (Irvine Scientific, California, USA) to the blastocyst stage and cryopreserved by using a Kitazato vitrification kit (Kitazato, Japan).

**Figure 2:**
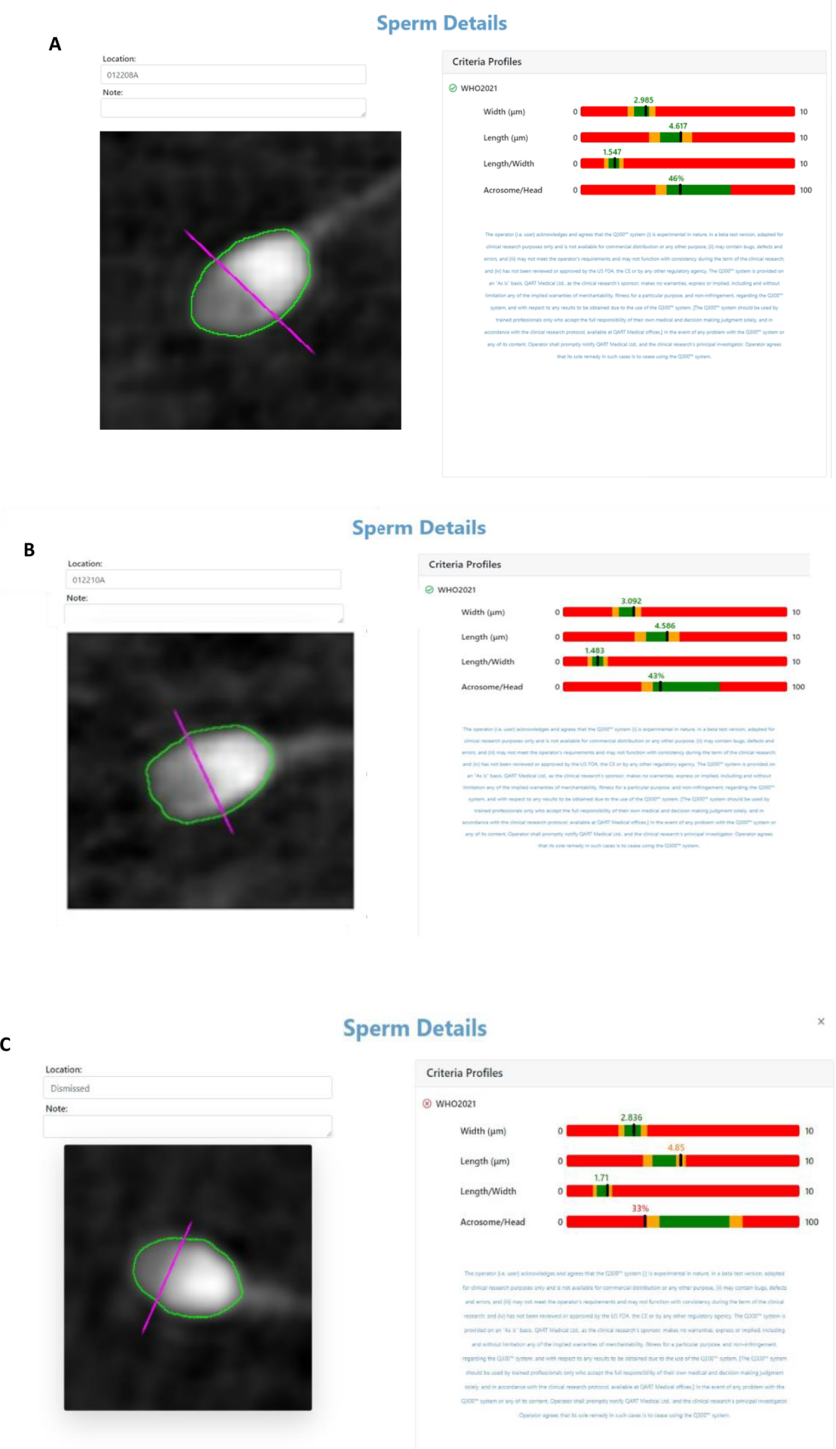
**(A,B)** Q300 results screen of the complaint sperm cells according to WHO criteria injected into the transferred embryos. **(C)** Sperm cell that did not pass according to WHO criteria.

The two embryos transferred were created using sperm cells deemed “fully morphologically compliant with WHO2021 criteria” (11) by the Q300 device (Figures 2A,B)The sperm cell example that failed to meet the WHO criteria is attached (Figure 2C).

**Figure 3:**
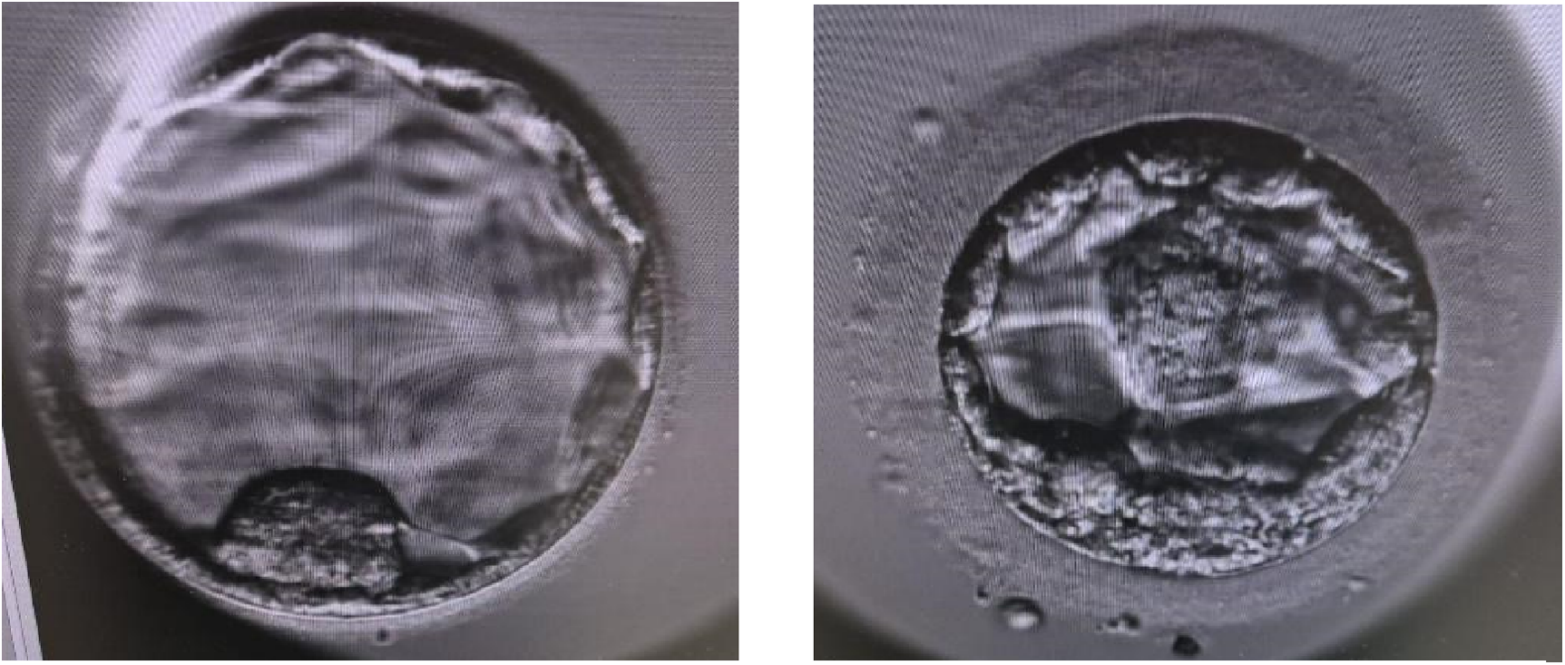
Blastocysts that were developed by using Q300 device and transferred.

Two blastocysts (Figure 3) were thawed using a Kitazato warming kit (Kitazato, Japan) and transferred to the dish containing Continuous Single Culture-NX Complete medium (CSCM -NXC) (Irvine Scientific, California, USA). Embryo transfer was performed using transabdominal ultrasound guidance employing a soft transfer catheter (Wallace, Cooper Surgical, USA).

The embryo transfer of two blastocysts was performed during the subsequent natural menstrual cycle. Following spontaneous menstruation, the patient was monitored by serial ultrasound for endometrial thickness, follicular development, and measurement of LH and progesterone levels, until a rise in LH level was observed corresponding to a day prior to OPU/ovulation. On the following day, progesterone luteal support was started with daily 800 mg micronized progesterone soft gel vaginal capsules (Utrogestan, Besins, Iscovesco, C.T.S., Petach Tikva, Israel) in two divided doses and Dydrogesterone (Duphaston, Abbott Medical Laboratories LTD, Israel) PO 30 mg in three divided doses. The patient also received two additional injections, one of recombinant hCG (250 mcg) (Ovidrel® Prefilled Syringe, Merck KGaA, Darmstadt, Germany) and the other of GnRH-agonist (triptorelin 0.1 mg), on days 3 and 7 from the beginning of progesterone as described by Orvieto et al. (14).

**Figure 4:**
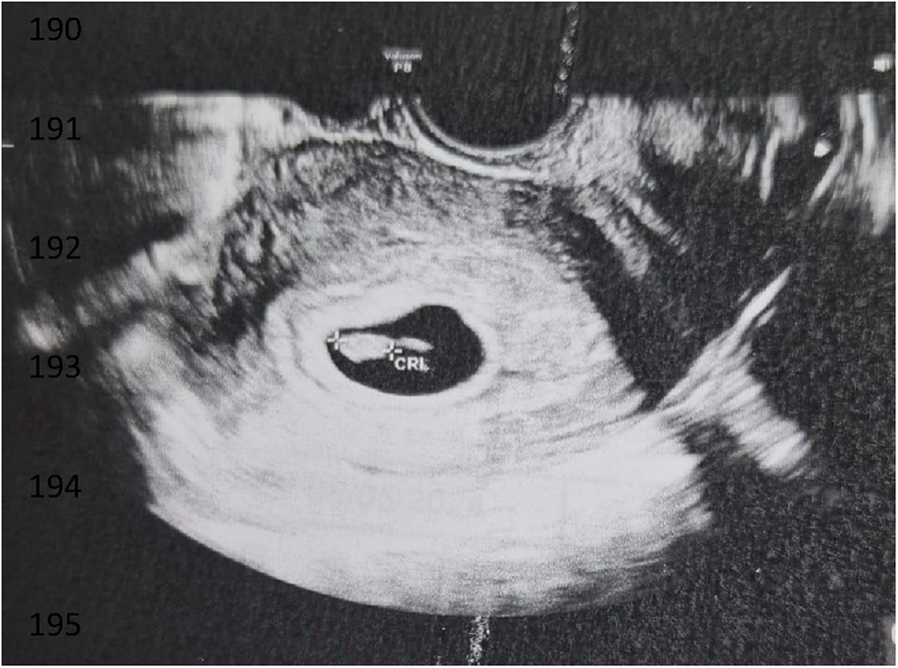
Ultrasound scan performed at 7 weeks of gestation.

Three weeks after the embryo transfer, the first scan was performed, revealing one amniotic sac with a fetal heartbeat (refer to Figure 4). The pregnancy ended successfully with the delivery of a healthy baby.

## Discussion

Infertility affects a significant proportion of couples worldwide, with male factor infertility contributing substantially to the challenges faced during conception (15). Assisted reproductive technologies, including IVF, have revolutionized fertility treatments by offering solutions to various fertility issues. Despite these advancements, recurrent IVF failures remain a distressing reality for many couples.

The male partner in this case, had severe OTA, significantly limiting the availability of morphologically normal sperm. Traditional sperm selection methods that need to be standardized to achieve uniformity and concomitance among embryologists (6) had not resulted in successful pregnancies in the previous 15 IVF attempts.

Numerous studies have shown that sperm morphology is an important predictor of fertilization and pregnancy rates during ICSI (16). The Q300 technology provided a more precise and objective assessment of sperm morphology, allowing the embryologist to select the most viable sperm cells with higher accuracy than conventional methods (8–10). This technique (Q300) enhances sperm selection based on the internal organelles' measurements (virtual staining) and objective automatic assessment of the cells according to the WHO 2021 guidelines, without chemical staining. By focusing on selecting the healthiest and most viable sperm for fertilization, this technology aims to improve embryo quality and increase the chances of successful implantation.

The outcome of implementing Q300 technology was remarkably positive for our patient couple resulted in the formation of high-quality blastocysts (grades 4AA and G2) according to the Gardner grading system (13) that were not achieved in prior cycles using standard sperm selection techniques. After years of disappointment and multiple failed IVF attempts, they achieved a successful pregnancy that concluded with a delivery. This highlights the potential of innovative technologies to address specific barriers to conception, especially in cases where conventional methods have proven ineffective.

Furthermore, the successful outcome of this case underscores the importance of personalized medicine in fertility treatments. Tailoring interventions to individual patient profiles, including sperm quality assessments, can significantly impact treatment success rates. The ability to adapt and integrate emerging technologies into clinical practice expands the repertoire of tools available to fertility specialists, offering renewed hope to couples facing infertility challenges. Furthermore, this new highly informative 3D imaging tool sets the basis for future AI-based sperm selection and might bring to new criteria and standardization in sperm analysis and selection.

It is essential to acknowledge the limitations of this report, including the single-case nature and the need for larger studies to validate the efficacy and reproducibility of this new sperm selection technology. Long-term follow-up of pregnancies achieved through such innovations will provide valuable insights into their safety and clinical outcomes.

## Conclusion

In conclusion, this case report illustrates the transformative potential of the Q300 technology in overcoming male factor infertility and achieving successful pregnancies in challenging cases. Continued research and clinical application of these advancements are crucial for advancing the field of infertility.

## Data Availability

The raw data supporting the conclusions of this article will be made available by the authors, without undue reservation.
